# Water-Soluble Bismuth(III) Polynuclear Tyrosinehydroximate Metallamacrocyclic Complex: Structural Parallels to Lanthanide Metallacrowns

**DOI:** 10.3390/molecules25194379

**Published:** 2020-09-23

**Authors:** Marina A. Katkova, Grigory Y. Zhigulin, Roman V. Rumyantcev, Galina S. Zabrodina, Vladimir R. Shayapov, Maxim N. Sokolov, Sergey Y. Ketkov

**Affiliations:** 1 G.A. Razuvaev Institute of Organometallic Chemistry RAS, 603950 Nizhny Novgorod, Russia; gzhigulin@gmail.com (G.Y.Z.); romanrum@iomc.ras.ru (R.V.R.); kudgs@mail.ru (G.S.Z.); sketkov@iomc.ras.ru (S.Y.K.); 2 Nikolaev Institute of Inorganic Chemistry SB RAS, 630090 Novosibirsk, Russia; vladimir@niic.nsc.ru (V.R.S.); caesar@niic.nsc.ru (M.N.S.); 3 Chemistry Department, Kazan (Volga Region) Federal University, 420097 Kazan, Russia

**Keywords:** bismuth(III), lanthanide(III), tyrosinehydroximate, polynuclear metallamacrocyclic complex, metallacrown, X-ray structure, DFT calculation

## Abstract

Recently there has been a great deal of interest and associated research into aspects of the coordination chemistry of lanthanides and bismuth—elements that show intriguing common features. This work focuses on the synthesis and characterization of a novel bismuth(III) polynuclear metallamacrocyclic complex derived from aminohydroxamic acid, in order to compare the coordination ability of Bi^3+^ with the similarly sized La^3+^ ions. A polynuclear tyrosinehydroximate Bi(OH)[15-MC_Cu(II)Tyrha_-5](NO_3_)_2_ (**1**) was obtained according to the synthetic routes previously described for water-soluble Ln(III)-Cu(II) 15-MC-5 metallacrowns. Correlations between structural parameters of Bi(III) and Ln(III) complexes were analyzed. DFT calculations confirmed the similarity between molecular structures of the model bismuth(III) and lanthanum(III) tyrosinehydroximate 15-metallacrowns-5. Analysis of the electronic structures revealed, however, stronger donor-acceptor interactions between the central ion and the metallamacrocycle in the case of the lanthanum analogue. Thermochromic properties of **1** were studied.

## 1. Introduction

Bismuth(III) complexes have received increased interest due to their high effectiveness in eradication of *Helicobacter pylori*, and also as potential antimicrobial and anti-leishmanial agents [1,2,3]. Up to now, many coordination bismuth(III) compounds with a large variety of ligands and coordination environments had been synthesized and explored in biomedical applications [4,5,6,7,8]. Recent interest in the use of alpha-emitting ^213^Bi in nuclear medicine requires efficient Bi complexation, preferably within a macrocyclic cavity [9,10]. It is not surprising that research on the synthesis of biologically important ligands and their water-soluble complexes has received significant attention [11,12,13,14,15,16,17]. However, the chemistry of Bi(III) explored in this purpose to date is rather scarce, and the study of bismuth complexes in aqueous solutions is often difficult.

Bismuth is the heaviest stable element and is recognized as a relatively low toxic metal, although conversely, it sits in the Periodic table among the most toxic and radioactive elements including mercury, thallium, lead, and polonium [18]. Additionally, within group 15, antimony and arsenic compounds are generally of high toxic nature, whereas those of bismuth exhibit a significantly reduced toxicity [19]. This behavior is probably caused by the unique electronic structure of the Bi^3+^ ion. The ground-state electron configuration of bismuth [Xe]4f^14^5d^10^6s^2^6p^3^ is particularly stable, and the three 6p electrons are responsible for bond formation on coordination [20]. Thus, in the numerous of Bi-containing complexes, bismuth exhibits the oxidation state of 3+. Noteworthy, the Shannon ionic radii of Bi^3+^ (1.03 and 1.17Å for coordination numbers of 6 and 8, respectively) are similar to those of La^3+^ (1.032 and 1.16Å, respectively) [21]. Consequently, in the first approximation based on electrostatic and steric factors, analogous complexes of bismuth and the lanthanides could adopt similar coordination geometries [22]. Sometimes, however, this is not the case. Specifically, Evans et al. described the synthetic and structural results in organobismuth chemistry that allow comparisons between the coordination geometries of bismuth and the lanthanides. This study found that it is necessary to take into account that the coordination chemistry of bismuth can be influenced by a stereochemically active lone pair, and bismuth has much higher Pauling electronegativity (1.9) than that of lanthanides (1.10–1.25) [22]. These similarities and differences certainly deserve more research efforts, particularly in the exploration of novel structural motifs of more developed lanthanide chemistry in order to design and synthesize new Bi(III) compounds.

**Scheme 1:**
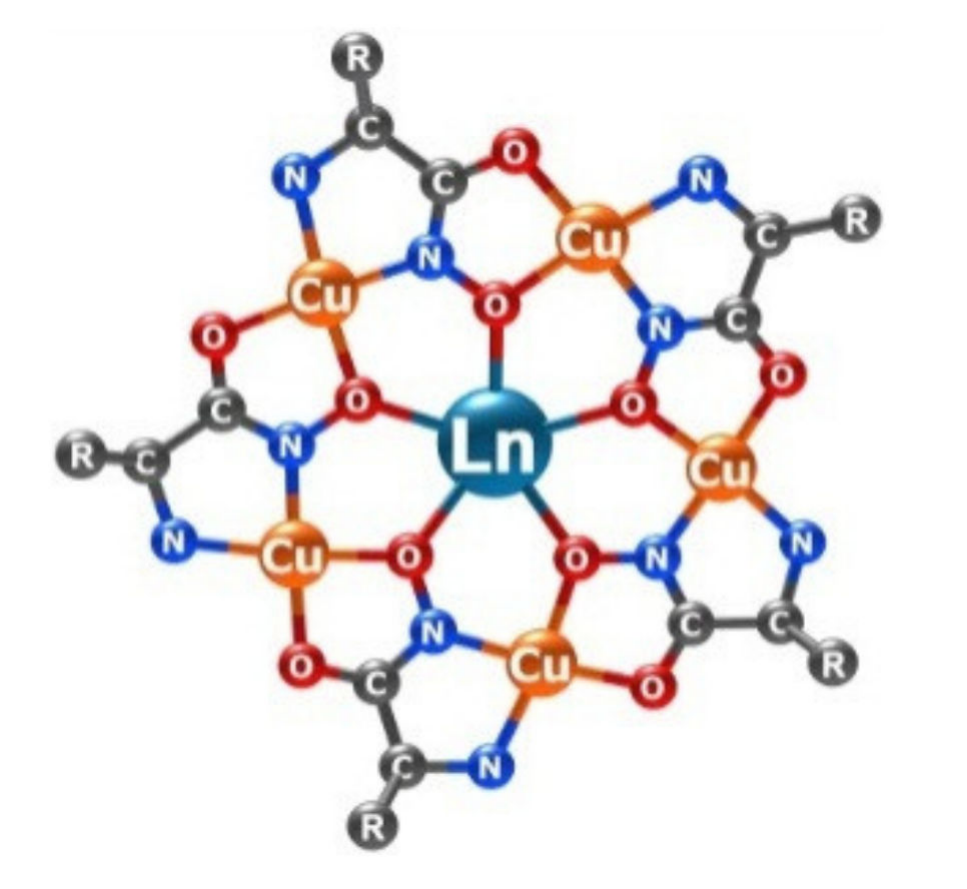
Schematic representation of Ln(III)-Cu(II) [15MC-5]. Hydrogen atoms, additional anions, and solvate molecules are omitted for the sake of simplicity.

Herein, to develop the next generation of coordination Bi(III) complexes with biologically important ligands, we considered polynuclear aminohydroximate Ln(III)-Cu(II) complexes, which belong to a family of 15-MC-5 metallacrowns. The popularity of Ln(III)-Cu(II) 15-MC-5 complexes is largely due to their fascinating architectures and potential applications [23,24,25,26,27,28]. These complexes present snowflake-like structures (Scheme 1) with the neutral ring consisting of five [Cu(II)-N-O] repeating units and the five hydroximate oxygen atoms encapsulating a Ln^3+^ ion within the central cavity.

A relevant aspect of this structure is that the ligand scaffold prefers early lanthanides characterized by the same charge and similar ionic radii as Bi^3+^. Recently, we have demonstrated the first metallamacrocyclic 15-MC-5 complex constructed from Cu^2+^ and Bi^3+^ metal centers and pyrazinohydroxamic moieties [29]. The single-crystal structure reveals the classic metallamacrocyclic 15-MC-5 configuration. The Bi^3+^ ion is located at the center of the 15-MC-5 ring consisting of five [Cu(II)-N-O] repeating units. Following our interest in water-soluble Ln(III)-Cu(II) 15-MC-5 metallacrowns with aminohydroximate ligands [30,31,32,33,34], we describe here a general synthetic approach and comparable characterization of a new water-soluble metallamacrocyclic Bi(III)-Cu(II) 15-MC-5 complex derived from tyrosinehydroxamic acid.

## 2. Results and Discussion

### 2.1. Synthesis and Spectroscopic Aspects

**Scheme 2:**
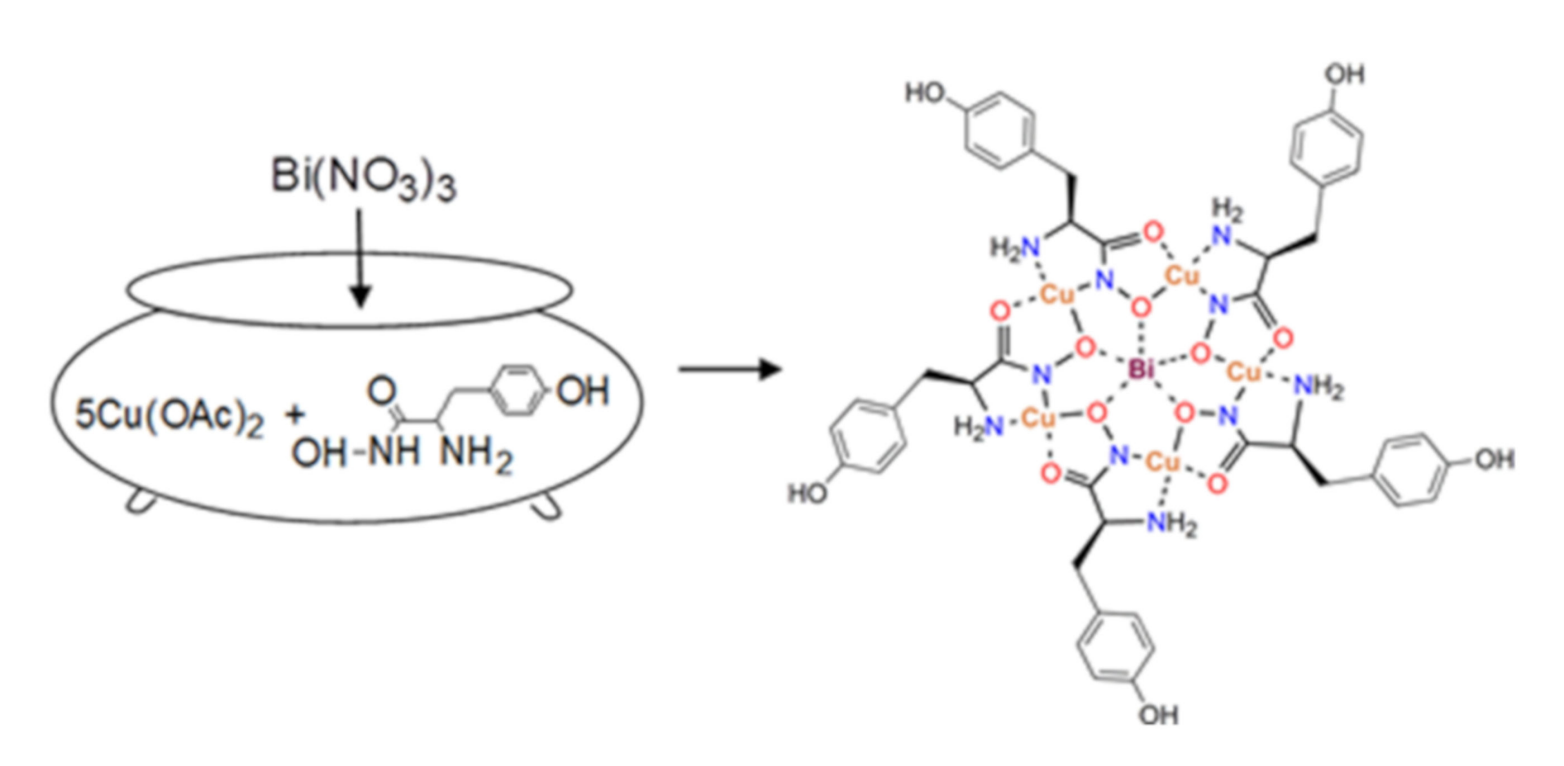
Schematic representation of the synthesis of complex **1**.

Following a previously described synthetic procedure for water-soluble Ln(III)-Cu(II) 15-MC-5 metallacrowns [30,31] we employed the most frequently used two-step methodology with some modification. In the first step, α-tyrosinehydroxamic acid and Cu(CH_3_COO)_2_ were mixed in water and, in the second step, Bi(NO_3_)_3_ was added to this solution (Scheme 2).

**Figure 1:**
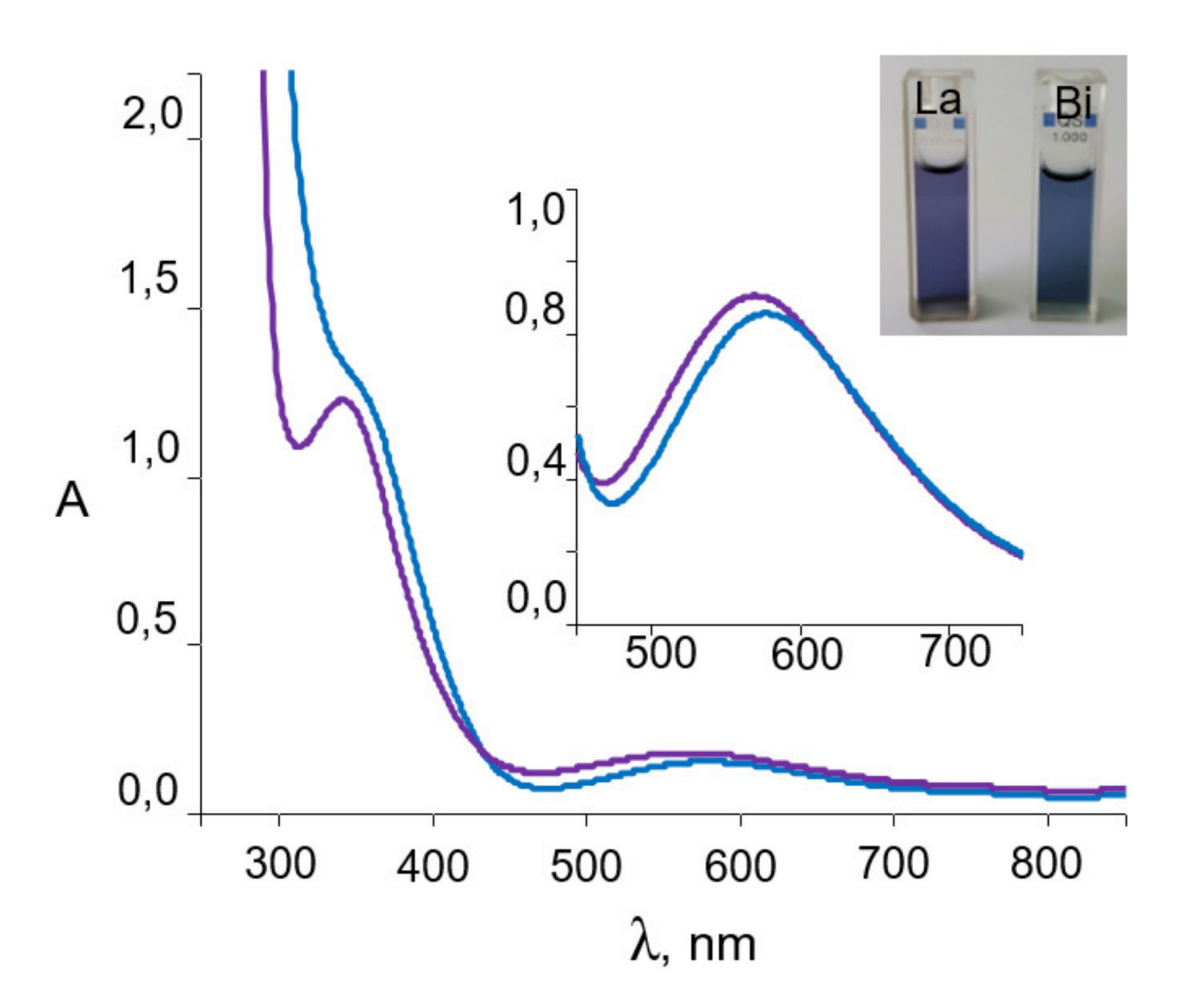
UV-vis spectra of the aqueous solution of **1** (blue) and the analogous La complex (violet). Inset: Spectra at 575 nm (2.3 × 10^−3^ M).

It can be seen (Figure 1) that the absorption spectra of the resulting complex **1** are similar to those of La complex. In the visible region a broad band with the maximum at 575 nm (ε = 395 M^−1^ cm^−1^), assigned to Cu(II) d-d transition, is responsible for the characteristic dark blue color, which is observed in all previously reported water-soluble Ln(III)-Cu(II) aminohydroximate complexes [23,24,30,31].

The yield of isolated crystalline **1** was 32%, in contrast to 85% of the lanthanum analogue, because of hydrolysis of Bi^3+^ in water. Crystals of **1** suitable for the single-crystal X-ray diffraction experiment were obtained by recrystallization from water. It is worth mentioning that the stability of complex **1** in aqueous solutions was confirmed using UV-vis spectroscopy by recording absorption spectra at a pH value of about 7, important for biological studies (pH 6.8). No absorbance changes even after several days were recorded in either the intensity or the position of the absorption bands. Nevertheless, in contrast to the lanthanum complexes, in alkaline medium (greater than pH 8) the rapid hydrolysis of **1** occurs with the formation of an amorphous pale precipitate.

### 2.2. Thermochromic Properties

**Figure 2:**
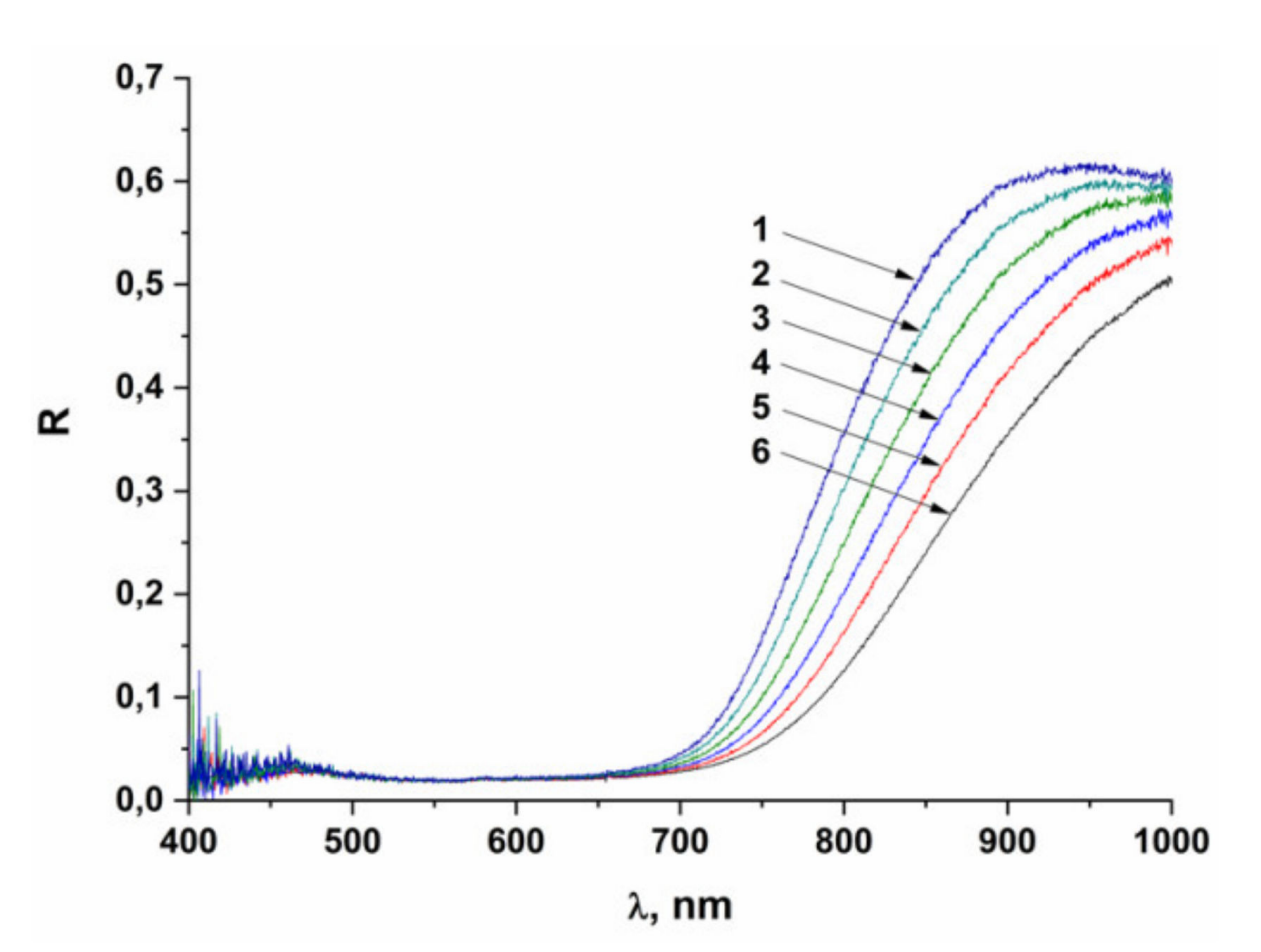
Diffuse reflection spectra of the sample obtained at temperatures: −175 °C (**1**), −141 °C (**2**), −100 °C (**3**), −58 °C (**4**), −21 °C (**5**), +21 °C (**6**).

Compound **1** exhibits interesting low-temperature thermochromism. Diffuse reflection spectra recorded in the range from −175 to +21 °C are shown in Figure 2. They show a monotonous shift toward near the IR region.

**Figure 3:**
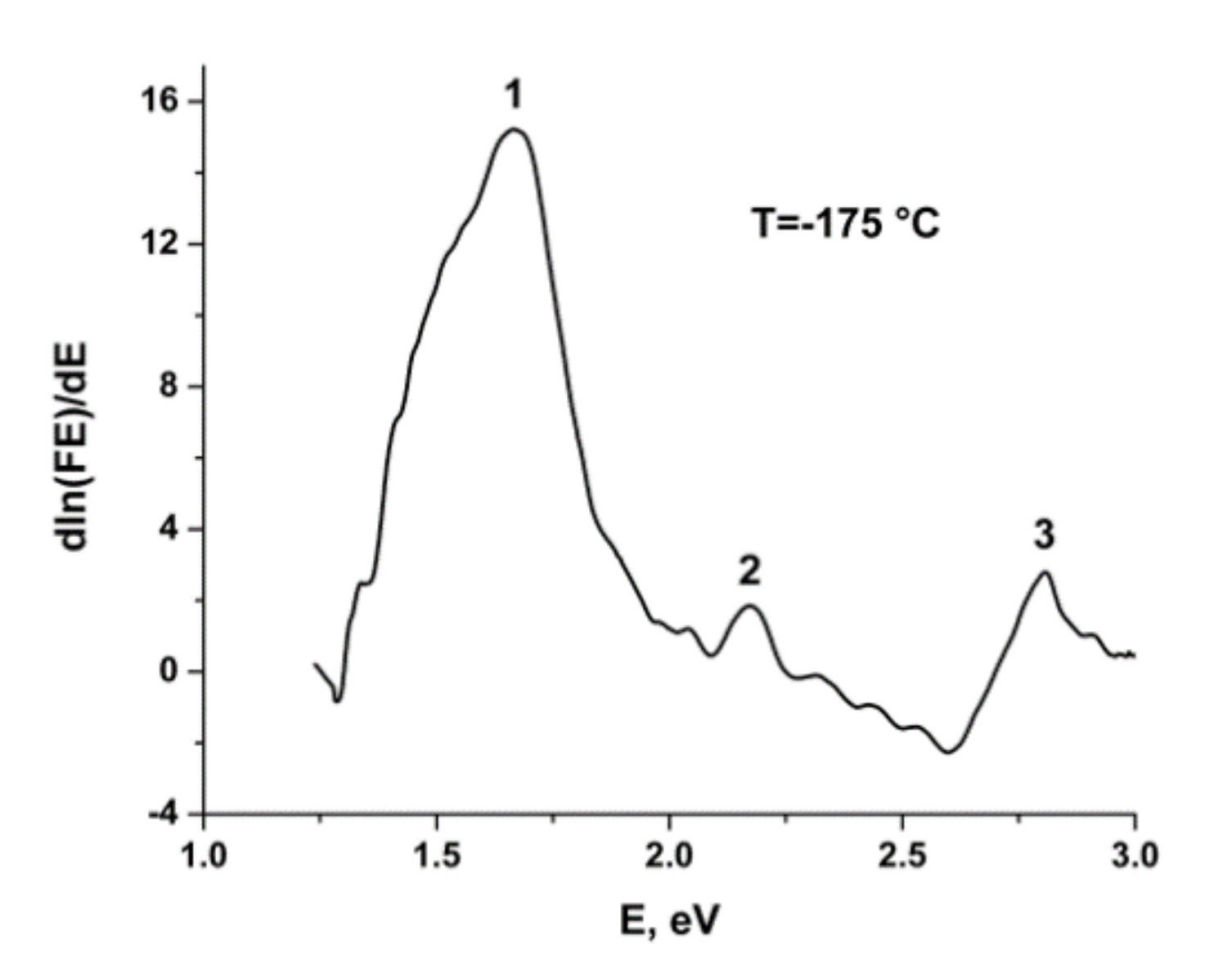
Dependence of dln(F(R)·E)/dE on energy at −175 °C.

To determine the absorption value from the diffuse reflection spectra, the spectra of the Kubelka-Munk function F(R) were calculated:

*F*(*R*) = (1 − *R*)^2^/(2*R*) (1)

where R is the diffuse reflection coefficient. The Kubelka-Munk function is directly proportional to the absorption coefficient k and inversely proportional to the scattering coefficient s of the powder, which is virtually independent on the wavelengths. Therefore, the shape of *F(R)* qualitatively approximates the absorption spectrum. The procedure described in [35] was applied to determine the value of the gap (E_g_) of interband electronic transitions. In the range of the electronic transition, the dependence of the absorption coefficient α on the energy E has the form:

α = *A*(*E* − *E*_g_)*^m^*/*E* (2)

where A and m are the constants of the electronic transition and E_g_ is its energy. In this paper we used the Kubelka-Munk function F(R) as an approximation for α. This can be transformed into the following equation:

*d*ln(α*E*)/*dE* = *m*/(*E* − *E*_g_) (3)

which provides a simple way to determine E_g_, since the value of E_g_ will correspond to the maximum of this function. As an example, the dependence obtained at −175 °C is given in Figure 3.

**Figure 4:**
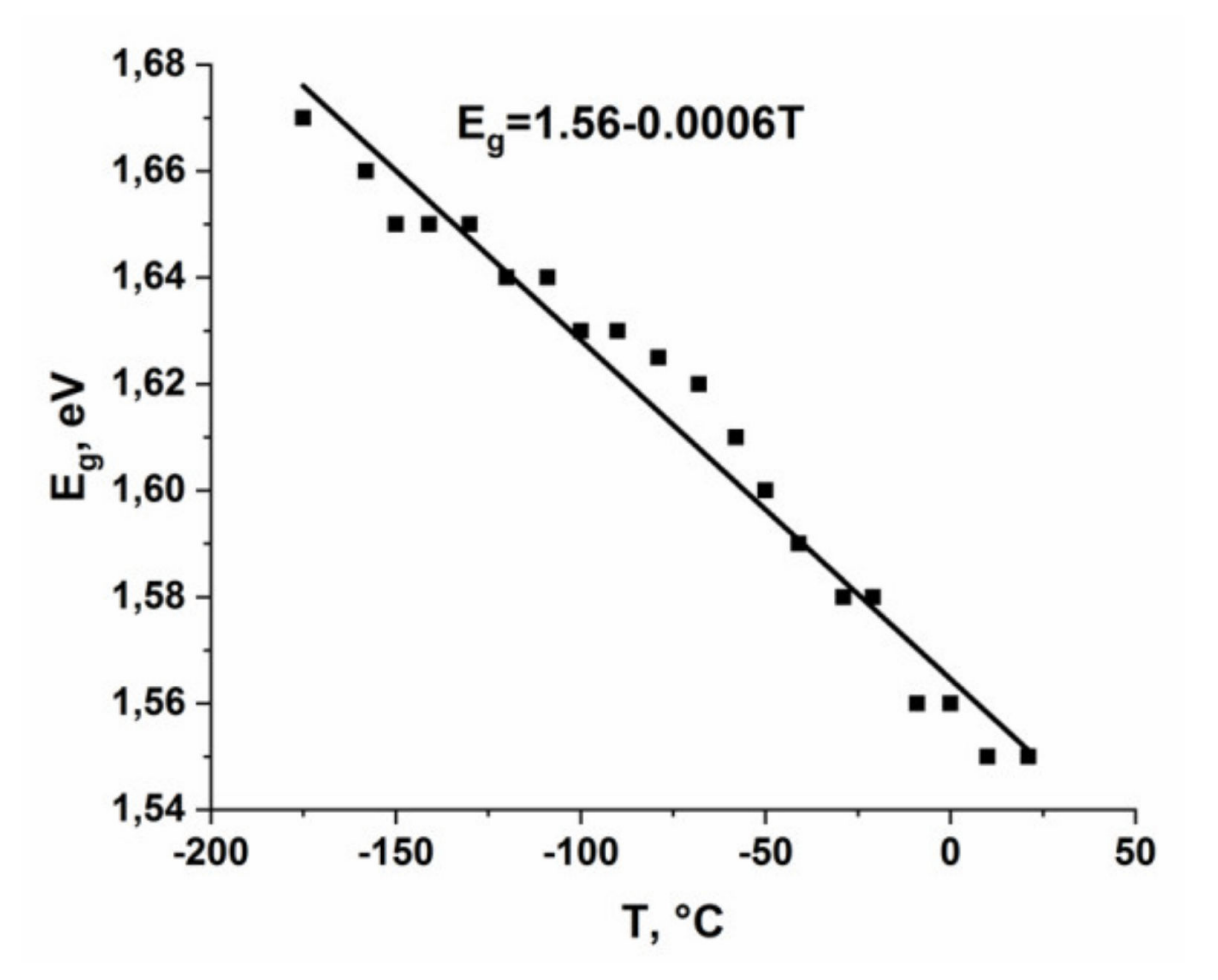
Temperature dependence of the d_z_^2^ → d_x_^2^_-y_^2^ transition energy (points) and its linear approximation (solid line). The graph shows the equation of a straight line that describes the experimental points in the best possible way.

The 1–3 maxima (Figure 3) can be assigned to the d_z_^2^ → d_x_^2^_-y_^2^ (742 nm), d_xy_ → d_x_^2^_-y_^2^ (571 nm), and d_xz,yz_ → d_x_^2^_-y_^2^ (446 nm) transitions, respectively. These d-d transitions are expected for the Cu^2+^ ion in square planar and square pyramidal coordination environment. The first transition formally corresponds to the band gap and is temperature-dependent (Figure 4).

Such a behavior might reflect a decrease of the bond lengths under cooling [36], which increases the antibonding d_x_^2^_-y_^2^ orbital energy and the width of the band gap (the HOMO-LUMO gap in an isolated complex). The temperature dependence of the other d-d- transitions is very weak. This can be caused by the π-admixture to the corresponding occupied d orbitals (they are π *-antibonding), which also results in an increase of their energies on cooling and reduces the band gap, therefore off-setting the influence of the raising d_x_^2^_-y_^2^ energy on the electronic transitions.

### 2.3. Structural Aspects: X-Ray Crystal Structure

**Figure 5:**
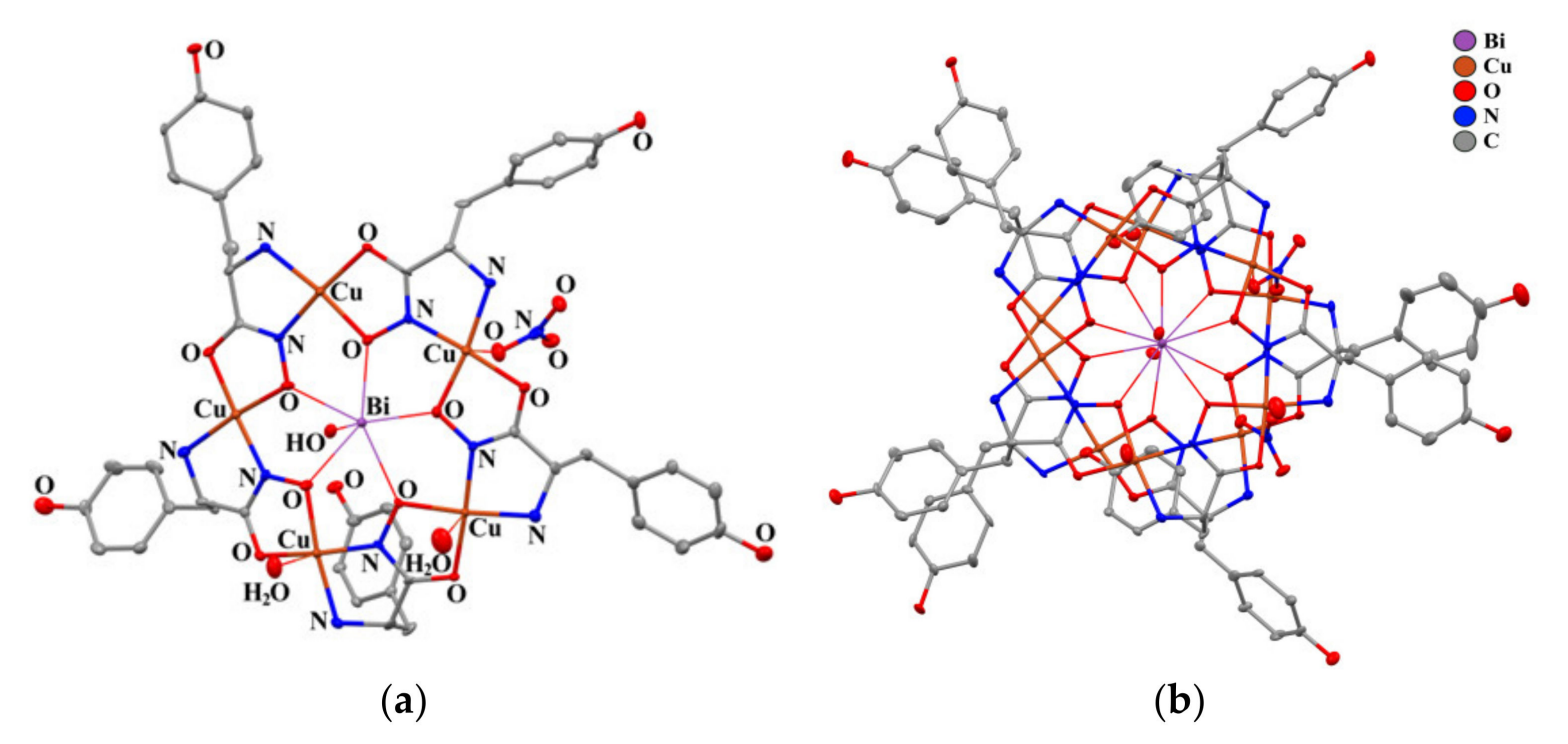
Top views of the structure of complex **1**: (**a**) monomer and (**b**) dimer pair shown looking down the pseudo fivefold axis. The thermal ellipsoids drawn are at the 30% probability level. All hydrogen atoms, uncoordinated NO_3_ˉ and solvate water molecules are omitted for clarity.

The X-ray crystal structure of **1** (Table 1) reveals the classic metallamacrocyclic 15-MC-5 configuration. The structure of **1** is depicted in Figure 5. The structural unit consists of two independent monomeric molecules (A and B) of the metallamacrocyclic Bi(III)–Cu(II) tyrosinehydroximate complex (Figure 5a). The molecules A and B have similar structures. The Bi atom is located at the center of the 15-MC_Cu(II)Tyrha_-5 ring, and is coordinated by five oxygen atoms of the ring in the equatorial plane. This Bi^3+^ complex **1** is expected to be isostructural to its La^3+^ analogue, since the ionic radii of both ions are comparable. However, according to the Cambridge Crystallographic Data Centre, no X-ray structural data on the La(III)-Cu(II) tyrosinehydroximate complexes have been reported thus far. Since the difference in the ionic radii between Bi^3+^ (CN = 6) and Gd^3+^ (CN = 8) is only 0.023 Å [21], the geometric characteristics of molecules **1** and known Gd(III)-Cu(II) tyrosinehydroximate complex are expectedly similar (Table 1), and the geometry of the Gd^3+^ complex can be used as reference. The crystallographic parameters of the gadolinium complex (a = 15.02060(10) Å, b = 28.9110(2) Å, c = 16.42480(10) Å, β = 103.9510(10)º, V = 6922.24(8) Å^3^, space group I2) [37] are also close to the corresponding values of crystal **1** (a = 14.7255(6) Å, b = 28.8331(12) Å, c = 16.2230(7) Å, β = 104.3559(16)º, V = 6672.9(5) Å^3^, space group P2(1)).

Note that the gadolinium atom in complex Gd(H_2_O)_3_[15-MC_Cu(II)Tyrha_-5](NO_3_)_3_ [37] is additionally coordinated by three oxygen atom of water molecules (two of which are located on one side in respect to Cu5 plane while the third on the other). In contrast to the lanthanide analogues, the Bi^3+^ ion is additionally coordinated by only one oxygen atom of the hydroxide anion in an apical position. The Bi-O distance (2.047(7), 2.106(8) Å) is in excellent agreement with the literature data on the compounds with the Bi-OH bonds [38,39,40]. The nearest water molecules are at the distance of 2.920(8) and 2.996(8) Å from Bi in the A and B molecules, respectively. These values significantly exceed the sum of the ionic radius of Bi^3+^ (1.03 Å) [21] and the van der Waals radius of the oxygen atom (1.55 Å) [41]. Thus, the coordination number of the bismuth atoms in both molecules is six, and the coordination environment is a pentagonal pyramid.

Two copper atoms additionally coordinate the water molecule in each of the independent molecules. One copper atom in each of the independent molecules has a strong interaction with the nitrate anion. Two NO_3_^-^ anions in the independent part of the cell are not coordinated to the metal atoms or the interaction is very weak. The closest distances of the oxygen atoms of these anions to the copper atoms are 2.924, 3.101, 3.157, and 3.267 Å.

The planes of the metallacrown rings are largely distorted. The average deviations of atoms from the plane are 0.18 and 0.16 Å for molecules A and B, respectively. It should be noted that the central bismuth atoms are localized practically in the plane of the metallacrown rings. The deviation of the Bi^3+^ ions from these planes are 0.036(A) and 0.184(B) Å.

**Figure 6:**
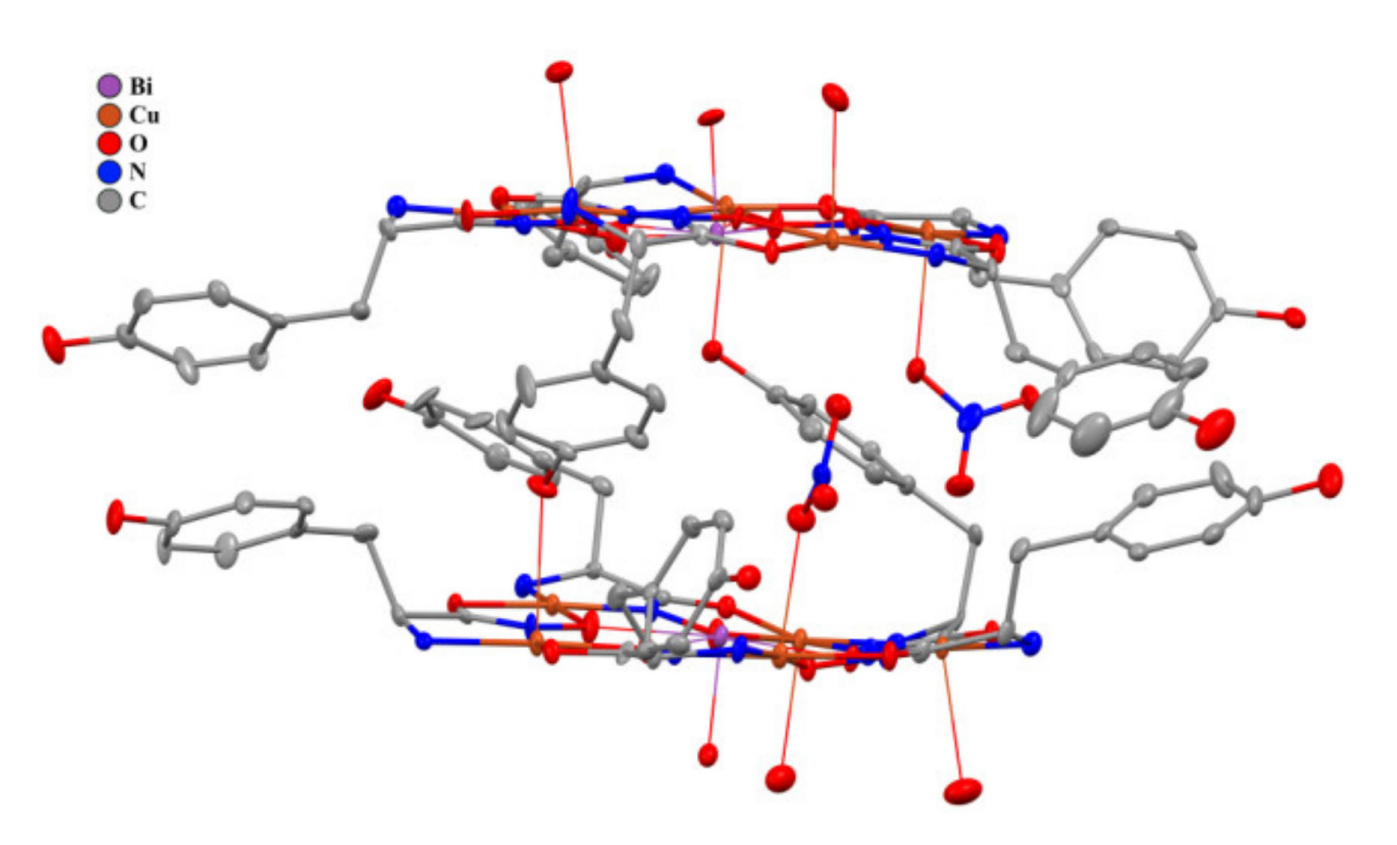
Side view of molecular structure of complex **1**. The thermal ellipsoids drawn are at the 30% probability level. All hydrogen atoms, uncoordinated NO_3_ˉ, and solvate water molecules are omitted for clarity.

A comparison of the related complex of bismuth with pyrazinohydroxamic acid [29] reveals significant differences in the crystal packing between these two compounds. The molecules of the bismuth complex with pyrazinohydroximate ligands form an infinite three-dimensional (3D) network due to N^...^H interactions and π^...^π stacking. Neighboring molecules are largely offset from each other and do not form dimeric motifs. In contrast, in complex **1**, one copper atom of the metallacrown ring is additionally coordinated by the hydroxyl oxygen atom of a neighboring molecule. The Cu-O(Tyrha) distances are 2.762(8) and 2.655(8) Å. Due to these interactions, the molecules of complex **1** form dimeric associates in the crystal (Figure 6). In addition, the coordinated NO_3_^-^ anions are arranged in such a way that one of the oxygen atoms is oriented toward the copper atom of the partner molecule in the dimeric species. The intermolecular distances of Cu-O(NO_3_ˉ) are 2.88 (1) and 2.95 (1) Å.

**Figure 7:**
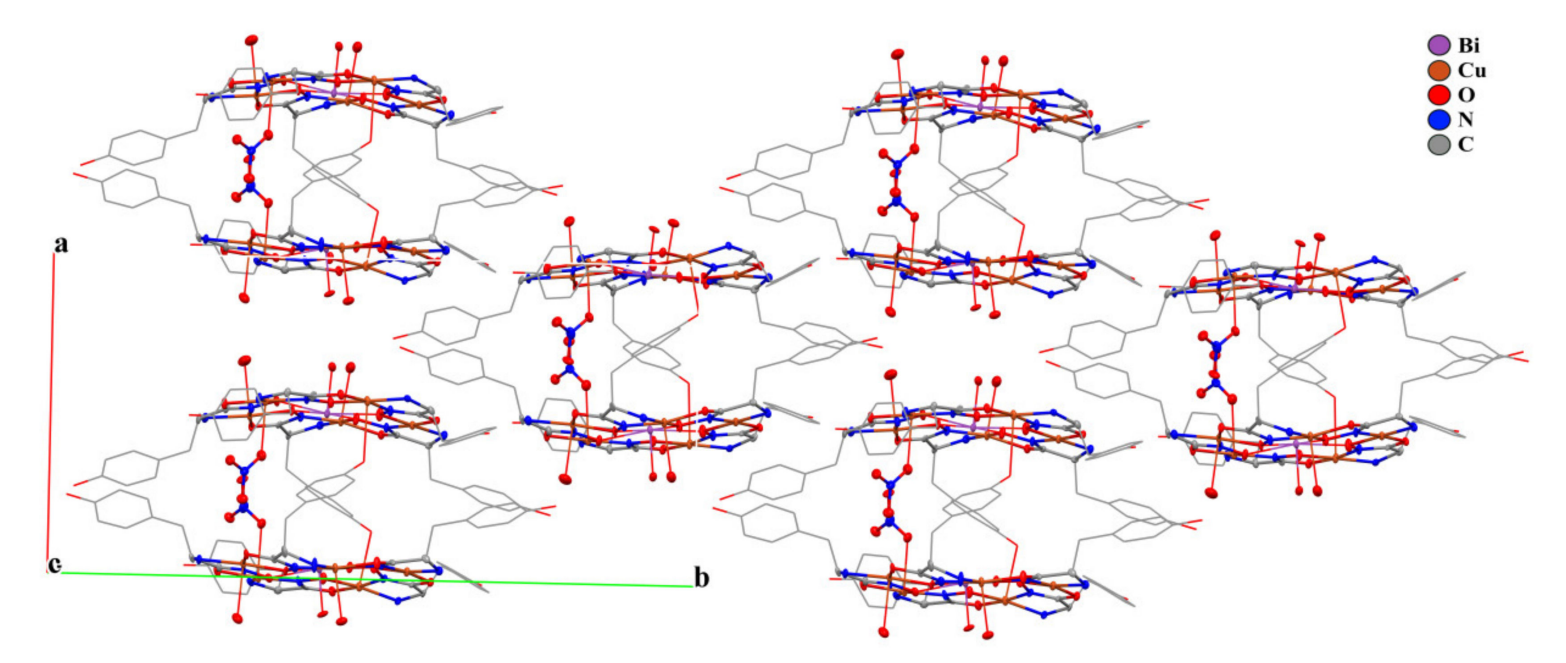
Fragment of crystal packing of complex **1**. All hydrogen atoms, uncoordinated NO_3_ˉ, and solvate water molecules are omitted for clarity.

Note that the crystal packing of complex **1** (Figure 7) is similar to that found in the gadolinium (III) tyrosinehydroximate complex [37]. Three water molecules are located inside the dimer cavity, both in complex **1** and in the gadolinium tyrosinehydroximate complex [37]. However, in the case of gadolinium, two water molecules are coordinated to Gd^3+^. Thus, the Gd-O bonds are almost perpendicular to the planes of the metallacrown rings. In turn, in complex **1**, the water molecules are significantly displaced from such an ideal position. This leads to the Bi...Bi distance in the dimers in **1** (7.17 Å) being much shorter than the corresponding Gd...Gd distance (8.39 Å). The distances between the copper atom and the hydroxyl group of tyrosinehydroximate ligand of the neighboring metallacrown are also significantly shorter in complex **1** (Table 1). In turn, the distance between dimer particles is shorter in the gadolinium complex (d(Gd…Gd) = 8.52 Å) [37] than the Bi...Bi distances in complex **1** (9.24 Å).

### 2.4. Theoretical Calculations

To shed some light on the exceptional bonding of **1**, DFT calculations were performed in this work. In contrast to the Bi complex bearing one OH^−^ group, the La 15-MC-5 complexes contain typically 4 H_2_O molecules coordinated at the axial positions of the La^3+^ ion. To provide comparable results when studying the bismuth(III) and lanthanum(III) ion influence on the metallamacrocyclic environment in the tyrosinehydroximate 15-metallacrowns-5, we analyzed the Bi and La model complexes without axial OH^−^ and H_2_O ligands [M(15-MC_Cu(II)Tyrha_-5)]^3+^ (M = Bi, La). Both structures were optimized at the scalar relativistic SR-PBE/rL2 level of DFT with the XRD atomic coordinates of complex **1** being used as the initial geometry approximation. Optimized interatomic distances of the [Bi(15-MC_Cu(II)Tyrha_-5)]^3+^ complex agree well with the experimental values of **1** (Table 1, Table 2 and Table 3). Moreover, the optimized structure of [La(15-MC_Cu(II)Tyrha_-5)]^3+^ appeared to be very similar to that of the bismuth derivative. The topological analysis of the electron density function ρ(**r**_c_) reveals negative values of the Laplacian ∇^2^ρ(**r**_c_) for covalent bonds at the (3,-1) critical points (Table 2) and positive ∇^2^ρ(**r**_c_) values for the Cu-N, Cu-O, Bi-O, and La-O coordination bonds (Table 3). In the tyrosinehydroximate ligands, adjacent C-N_im_ and C-O bonds are characterized by higher values of the electron density ρ(**r**_c_) and ellipticity ε at the (3,-1) critical points (0.358–0.362 a.u. and 0.099–0.289, respectively, Table 2). This indicates a π-delocalization among the N_im_, C, and O_carb_ atoms in the hydroximate chelates with the π-contributions into the C-N_im_ bonds being higher (ε = 0.277–0.289) than those for C-O (ε = 0.099–0.107). Ellipticity values of 0.175–0.195 calculated for the aromatic C-C bonds in the C_6_H_4_OH fragments are intermediate between those for C-N_im_ and C-O. Ellipticity values of 0.059–0.070 obtained for the N-O single bonds suggest some *p*,π-conjugation between the oxime oxygen lone pair and the delocalized π- system. Additionally, *p*,π-conjugation is expected to occur for C-O(H) bonds in the substituents (ε = 0.077–0.078). The C-C bonds in the hydroximate chelates (1.517–1.523 Å) are shorter than typical single C-C bonds (1.54 Å). Taking into account calculated ellipticity values of 0.104–0.107 one can assume the σ,π-conjugation (hyperconjugation) effect. Such interaction is also inherent in the single C-C_ring_ bonds (1.508–1.510 Å) between the H_2_C and C_6_H_4_OH fragments of the substituents (ε = 0.043–0.049). For comparison: C-C_R_ bonds between the hydroximate chelates and the substituents without σ,π-conjugation are characterized by the 1.547–1.552 Å lengths and ellipticity values close to zero (0.018–0.021).

Among the copper(II) coordination bonds the interactions with the imine nitrogen atoms of the tyrosinehydroximate ligands are estimated as the strongest ones with calculated energies of 59.9–61.3 kcal/mol (Table 3). The more elongated contacts with the amine nitrogen atoms are characterized by the lower values of 35.4–37.9 kcal/mol. Energies of copper(II) bonds with the oxime and carbonyl oxygen atoms are closer: 35.9–40.4 and 46.3–47.0 kcal/mol, respectively. In general, the bonding situation in the hydroximate chelates of the [M(15-MC_Cu(II)Tyrha_-5)]^3+^ complexes is similar to that in the 15-metallacrowns-5 analyzed by us earlier with use of QTAIM in conjunction with the hybrid functionals τ-HCTHhyb [33] and M06 [29,42,43]. This makes the SR-PBE/rL2 calculations appropriate for description of the hydroximate 15-metallacrowns-5.

Calculated M-O_ox_ distances are very close: 2.433–2.442 Å in [Bi(15-MC_Cu(II)Tyrha_-5)]^3+^ and 2.432–2.438 Å in [La(15-MC_Cu(II)Tyrha_-5)]^3+^. These coordination bonds are characterized by the lower values of ρ(**r**_c_) and *E*_int_ (0.049–0.057 a.u. and 16.3–20.2 kcal/mol respectively, Table 3) as compared with Cu(II)-O (ρ(**r**_c_) = 0.080–0.094 a.u., *E*_int_ = 35.9–47.0 kcal/mol). Despite the small difference in the bond lengths the topological analysis reveals increase of the electron density at the (3,–1) critical points on going from Bi-O_ox_ (0.049–0.050 a.u.) to La-O_ox_ (0.056–0.057 a.u.) contacts. This is accompanied by increase of the interaction energies from 16.3–16.8 kcal/mol to 19.8–20.2 kcal/mol (or from 82.8 to 99.9 kcal/mol in total). Moreover, some decrease of the electron density occurs at the (3,–1) critical points of the adjacent Cu-O_ox_ and N-O bonds. In the [Bi(15-MC_Cu(II)Tyrha_-5)]^3+^ complex the calculated ρ(**r**_c_) values are 0.084–0.086 and 0.323–0.324 a.u. for Cu-O_ox_ and N-O bonds, respectively. In the [La(15-MC_Cu(II)Tyrha_-5)]^3+^ complex ρ(**r**_c_) = 0.080–0.082 a.u. for Cu-O_ox_ and 0.311–0.312 a.u. for N-O. Accordingly, energies of the Cu-O_ox_ interactions decrease slightly from 38.8–40.4 to 35.9–37.7 kcal/mol. In addition, one principal difference between the Cu(II)-O and M-O_ox_ interactions revealed by the topological analysis is a drastic inequality of the ellipticities. Corresponding values for M-O_ox_ contacts are significantly higher (0.182–0.241) than those for copper(II) bonds (0.014–0.074). Thus, a special role in the bonding between the oxime oxygen atoms and the central ion is played by the π-interactions. At the (3,–1) critical points of the La-O_ox_ bonds the π-contributions appeared to be higher than those for the Bi-O_ox_ contacts (ε = 0.240–0.241 and 0.182–0.184, respectively).

**Figure 8:**
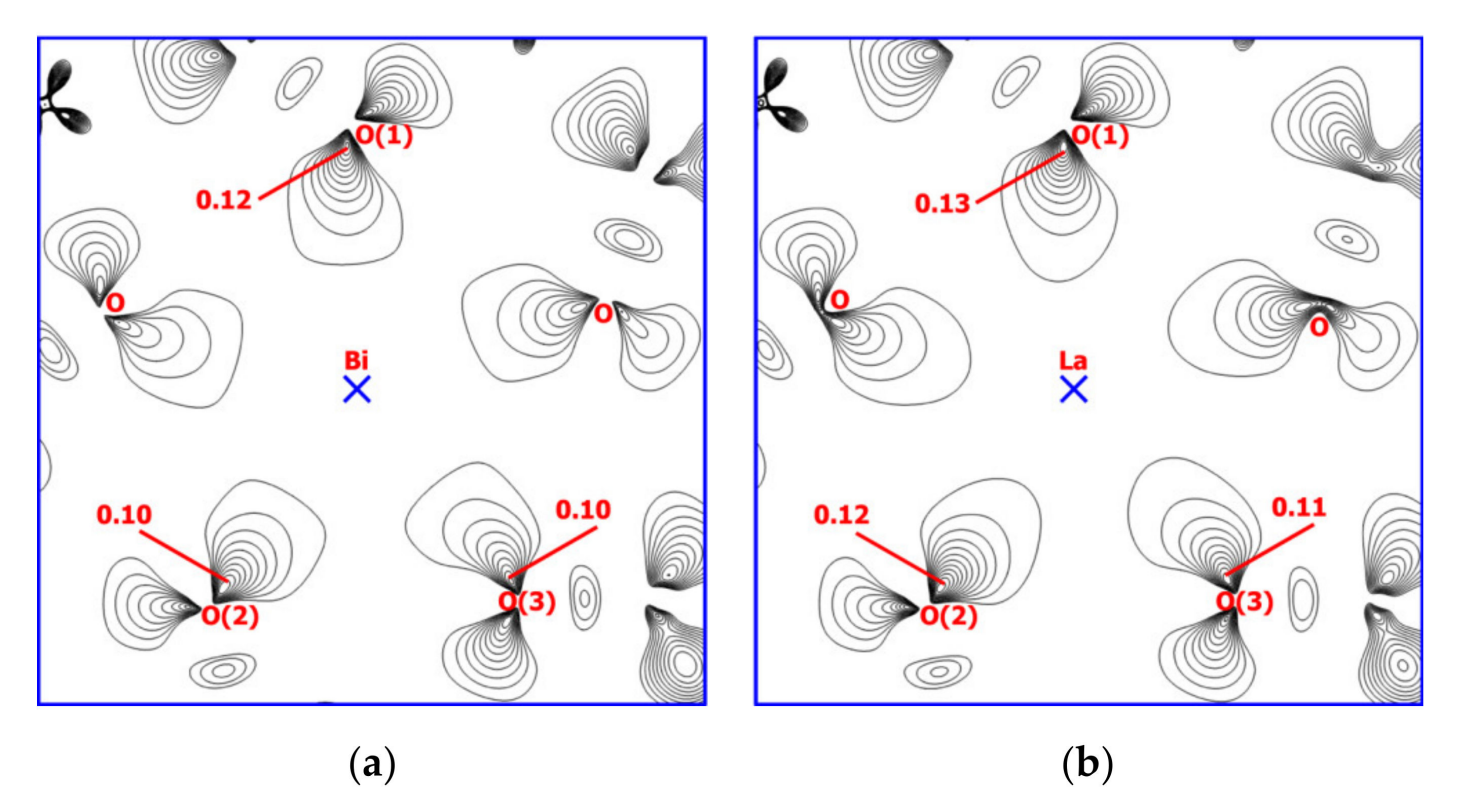
Deformation Electron Density maps in the plane of the oxime atoms O(1), O(2), and O(3): (**a**) For [Bi(15-MC_Cu(II)Tyrha_-5)]^3+^; (**b**) For [La(15-MC_Cu(II)Tyrha_-5)]^3+^. The contour lines start from 0.01 a.u. with step of 0.01 a.u. The maps are built at the same scale. The level of DFT calculations is PBE/x2c-TZVPall//SR-PBE/rL2.

**Figure 9:**
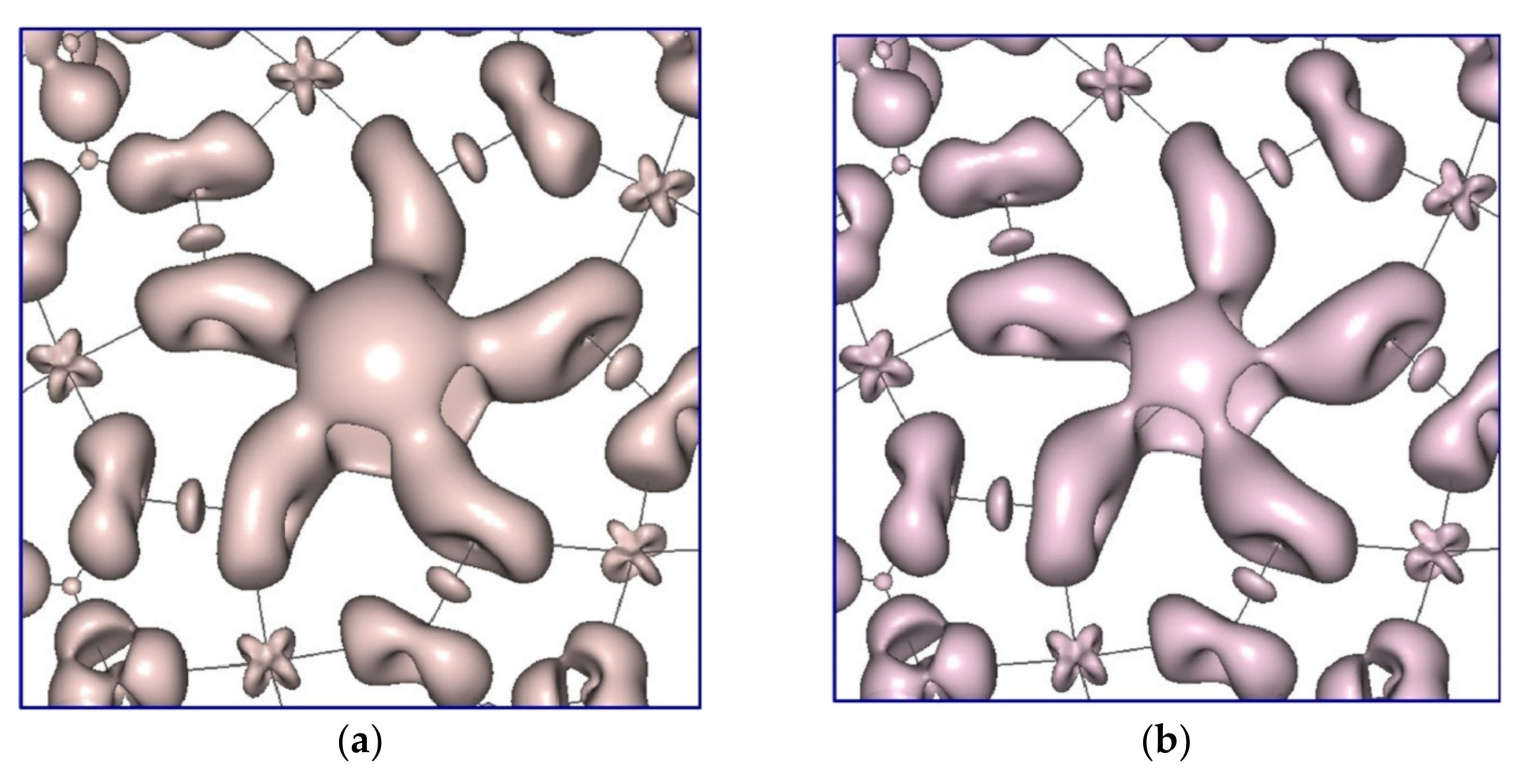
Deformation Electron Density isosurfaces at 0.01 a.u. in the region of the central ion and the five oxime oxygen atoms: (**a**) for [Bi(15-MC_Cu(II)Tyrha_-5)]^3+^; (**b**) for [La(15-MC_Cu(II)Tyrha_-5)]^3+^. The isosurfaces are built at the same scale. The level of DFT calculations is PBE/x2c-TZVPall//SR-PBE/rL2.

In the plane of the oxime oxygen atoms O(1), O(2), and O(3) distribution of the Deformation Electron Density (DED) investigated at the PBE/x2c-TZVPall//SR-PBE/rL2 level reveals some stretch of the lone pairs toward the La^3+^ ion unlike Bi^3+^ (Figure 8). The flattened shape of the oxygen lone pairs in [Bi(15-MC_Cu(II)Tyrha_-5)]^3+^ appears to be associated with higher σ-contribution into the Bi-O_ox_ interactions. Meanwhile, the larger density accumulation at the axial positions of the Bi^3+^ ion as compared with La^3+^ is clearly demonstrated by the DED isosurface together with the π-interactions (Figure 9). This results in the less effective positive charge on the central Bi ion and the less negative charges on the oxime oxygen atoms. The Mulliken charges calculated at the PBE/x2c-TZVPall//SR-PBE/rL2 level are +1.022*e* on Bi and –(0.436–0.434)*e* on the oxime oxygen atoms in [Bi(15-MC_Cu(II)Tyrha_-5)]^3+^; +1.601*e* on La and –(0.533–0.530)*e* on the oxime oxygen atoms in [La(15-MC_Cu(II)Tyrha_-5)]^3+^. The Mulliken charges calculated at the SR-PBE/rL2 level are +1.008*e* on Bi and –(0.398–0.397)*e* on the oxime oxygen atoms in [Bi(15-MC_Cu(II)Tyrha_-5)]^3+^; +1.592*e* on La and –(0.439–0.437)*e* on the oxime oxygen atoms in [La(15-MC_Cu(II)Tyrha_-5)]^3+^. The increase in the positive charge of the central ion on going from [Bi(15-MC_Cu(II)Tyrha_-5)]^3+^ to [La(15-MC_Cu(II)Tyrha_-5)]^3+^ is also predicted by the Bader QT AIM approach at the PBE/x2c-TZVPall//SR-PBE/rL2 level (+1.911*e* on Bi and +2.041*e* on La). On the basis of our calculations, one can conclude that accumulation of the density at the axial positions of the Bi^3+^ ion together with its decreased positive charge prevents filling the Bi^3+^ coordination sphere by the solvent molecules. This leads to the more effective bonding with the HO^−^ negative ion in **1** (as well with Cl^−^ in the pyrazinohydroximate complex [29]). As a result, the Bi^3+^ ion in the complex is characterized by the lower coordination number as compared with the La(III) 15-metallacrowns-5 where the La^3+^ central ion bears four H_2_O molecules at the axial positions [33]. Accordingly, the higher DED accumulation in the axial positions of the Bi^3+^ ion induces weakening of the Bi-O_ox_ donor-acceptor interactions in comparison with the La-O_ox_ contacts that has been described above.

## 3. Materials and Methods

All chemicals were reagent-grade and were used as received from Sigma Aldrich without further purification. The C, H, and N elemental analyses were performed by the Microanalytical laboratory of IOMC on Euro EA 3000 Elemental Analyzer. Electronic absorption spectra were recorded with the Perkin Elmer Lambda 25 UV/Vis spectrophotometer at room temperature, at 200–1100 nm. IR spectra were obtained on a Perkin Elmer 577 spectrometer and recorded from 4000 to 450 cm^−1^ as a Nujol mull on KBr plates.^1^H NMR spectra were recorded on BrukerAvance III 400 MHz spectrometer. Samples were dissolved in high purity D_2_O (Sigma Aldrich, St Louis, MO, USA), and the chemical shifts were referenced to the solvent peak. Diffuse reflection spectra were measured with a spectrophotometric system consisting of a Kolibri-2 spectrometer (VMK “Optoelectronika,” Novosibirsk, Russia), an FCR-7UV400-2-ME reflection/backscattering probe (Avantes, The Netherlands), and an AvaLight-DHS light source (Avantes, The Netherlands) [35]. The spectra were recorded in the 400–1000 nm range. BaSO_4_ powder was used as a reference for 100% reflection. Temperature measurements of the reflection spectra were performed from −175 °C to room temperature at 20 temperature points.

### 3.1. Synthesis

*Bi(OH)[15-MC_Cu(II)Tyrha_-5](NO_3_)_2_*(**1**). Bi(NO_3_)_3_⋅5H_2_O (0.097 g, 0.2 mmol) was added to a stirred solution of L-tyrosinehydroxamic acid (0.196 g, 1 mmol) and Cu(OAc)_2_⋅H_2_O (0.199 g, 1 mmol) in 50 mL of water. After stirring overnight and filtering, the solution was left to evaporate slowly. After two days dark-blue precipitate was formed and then filtered. The crystals were dissolved in hot water and recrystallized. Yield: 0.12 g (32%). Anal. calcd for C_90_H_154_Cu_10_Bi_2_N_24_O_70_ (3745.72): C 28.86, H 4.14, N 8.97. Found: C 28.92, H 4.11, N 8.95. IR (ν, cm^−1^): 3238 w, 1581 s, 1515 s, 1402 w, 1327 m, 1239 m, 1175 m, 1128 w, 1104 m, 1073 m, 1026 s, 965 w, 938 m, 852 w, 825 w, 808 s, 739 w, 648 w, 629 w, 598 m, 568 w, 543 w, 496 m. ^1^H NMR (D_2_O, 400 MHz, 298 K, δ_ppm_): 7.65 (br.s, 2H, C_6_H_4_); 7.88 (s, 2H, CH_2_); 8.9–9.5 (m, 2H, C_6_H_4_); 54.3 (s, 1H, CH).

### 3.2. X-ray Crystallographic Studies

The X-ray diffraction data for **1** were collected on Bruker D8 Quest diffractometer (graphite-monochromator, MoK_α_-radiation, ω-scan technique, λ = 0.71073 Å, T = 100(2) K). The intensity data were integrated by using the SAINT program [44]. The SADABS program [45] was used to perform area-detector scaling and absorption corrections. The structure was solved by dual method [46] and was refined on F^2^ using all reflections with the SHELXTL package [47]. All non-hydrogen atoms were refined anisotropically. Hydrogen atoms were placed in calculated positions and refined in the riding-model (U_iso_(H) = 1.5U_eq_(O) in OH-groups and U_iso_(H) = 1.2U_eq_(C, N) in other groups). There are 22.7 water molecules in the unit cell per molecule of the complex. For all water molecules and hydroxide anions in **1**, the hydrogen atoms were not located.

The crystal data for **1** (C_90_H_154_Bi_2_Cu_10_N_24_O_70_): monoclinic crystal system, space group P2_1_, unit cell dimensions: a = 14.7255(6) Å, b = 28.8331(12) Å, c = 16.2230(7) Å, β = 104.3559(16)º, V = 6672.9(5) Å^3^, Z = 2, d_calc._ = 1.864 gcm^–3^, μ = 4.294 mm^−1^, F(000) = 3756, Crystal size 0.21 × 0.10 × 0.06 mm^3^, 1.917 < θº < 24.999, reflections collected/unique = 68837/23130, R_int_ = 0.0504, R_1_ = 0.0448, *w*R^2^ = 0.0938 (I > 2s(I)), R_1_ = 0.0572, *w*R^2^ = 0.0972 (all data), S(F^2^) = 1.042, largest diff. peak and hole 3.034 and –1.495 *e*Å^–3^. CCDC 2009753 for **1** contains the supplementary crystallographic data for this paper. These data can also be obtained free of charge at www.ccdc.cam.ac.uk/structures from the Cambridge Crystallographic Data Centre.

### 3.3. Computational Methodology

Our quantum chemical investigations of the [M(15-MC_Cu(II)Tyrha_-5)]^3+^ complexes (M = Bi, La) are based on the Density Functional Theory (DFT), the topological analysis of the electron density function, and studies of the Deformation Electron Density (DED) distribution. Full geometry optimizations of the complexes were performed with the Priroda 15 [48,49] software employing the PBE functional [50] in conjunction with the four-component one-electron scalar relativistic (SR) approximation to the full Dirac equation where all spin-orbit terms are neglected. For all atoms we used the original all-electron relativistic correlation-consistent rL2 basis set of the triple-ζ polarized quality [51], which is an analogue of the well-known cc-pVTZ. The complexes were treated as high-spin sextet systems without symmetry constraints. For the SCF convergence criterion, a value of 10^–6^ was assigned. Tolerance on the gradient achieved in the geometry optimizations is 10^–5^. For accuracy of the integration grid, a value of 10^–8^ per atom was used. Harmonic vibrational frequencies were calculated to ensure the optimized stationary points to be local minima without negative eigenvalues. Assignment of the “print=+density” keyword in the Priroda 15 code allowed us to obtain values of the electron density ρ(**r**_c_) at the critical points and corresponding eigenvalues λ_1_, λ_2_, and λ_3_ of the hessian **A**(**r**_c_). Thus, Laplacian∇^2^ρ(**r**_c_) and ellipticity ε values at the critical points were calculated with the Equations (4) and (5) [52]:

∇^2^ρ(**r**_c_) = λ_1_ + λ_2_ + λ_3_ (4)

ε = λ_1_/λ_2_ − 1 (5)

One of the important applications of the topological analysis is the estimation of interatomic interaction energies (*E*_int_) on the basis of the Espinosa correlation [53]. Initially, Equation (6) was proposed for hydrogen bond energy calculations. Later, its usage was extended to coordination bonds in metal complexes [54,55]. We calculated values of the potential electron energy density *V*(**r**_c_) at the critical points with the Equation (7) based on the approximation of the kinetic electron energy density [56] and the virial theorem [52]:

*E*_int_ = −(1/2)*V*(**r**_c_) (6)

*V*(**r**_c_) = −(3/5)(3π^2^)^2/3^ρ(**r**_c_)^5/3^ − (1/12)∇^2^ρ(**r**_c_) (7)

Calculations of atomic charges by the Bader approach [52] were performed employing the Sculpt basin integration algorithm implemented in the AIMAll software [57]. The DED maps and isosurfaces were obtained with the Multiwfn code [58,59] using the wave functions computed with the Gaussian 09 package [60]. Corresponding Single Point Energy calculations were carried out for the molecular geometries optimized before with the Priroda 15 software. The PBE functional and the all-electron triple-ζ polarized x2c-TZVPall basis set [61] were applied at the Gaussian 09 calculations. Additionally, the ultrafine integration grid and default SCF convergence criteria without symmetry constraints were assigned (“Int = UltraFine” and “NoSymm” keywords in the Gaussian 09 code). The wave functions of the complexes were proven to be stable by the tests for the SCF solutions stability.

## 4. Conclusions

The identical charge and ionic radii of Bi^3+^ and La^3+^ cations open up the possibility for the development of Bi(III)-based metallacrowns. Based on this analogy, we have designed and isolated a novel example of Bi(III)-Cu(II) hydroximate metallamacrocyclic complex of the 15-MC-5 type. The single crystal X-ray diffraction measurements confirmed the classic metallamacrocyclic 15-MC-5 configuration with the neutral ring consisting of five [Cu(II)-N-O] repeating units, and the five hydroximate oxygen atoms encapsulating a Bi^3+^ ion within the central cavity. Scalar relativistic DFT investigations predict similarity between molecular structures of the model bismuth(III) and lanthanum(III) tyrosinehydroximate 15-metallacrowns-5. At the same time analysis of the electronic structures reveals stronger donor-acceptor interactions between the central ion and the metallamacrocycle in the case of the lanthanum analogue. The larger density accumulation at the axial positions in the pentagonal pyramid ligand environment around the Bi^3+^ ion explains the preferential coordination of the negatively charged OH^−^ ion instead of the solvent molecule, as well as the exceptional six-coordinated geometry. Coordination of both Bi(III) and La(III) ions is characterized by significant π-contributions to the interactions with the metallamacrocycle. To conclude, we have prepared a new interesting bismuth(III) metallacrown complex, which may not only enrich the structure diversity of both metallacrowns and bismuth coordination compounds, but also offers another approach to discovery of new polynuclear metallamacrocyclic complexes with unconventional structures and properties.

## Figures and Tables

**Table 1:** Comparison of X-ray structural parameters of complex **1** with Bi(Cl)(H_2_O)[15-MC_Cu(II)Pyzha_-5](NO_3_)_2_ [29] and Gd(H_2_O)_3_[15-MC_Cu(II)Tyrha_-5](NO_3_)_3_ complexes [37].

Distances [A] and angles [°]	1A (M = Bi)	1B (M = Bi)	Bi(Cl)(H_2_O)[15- MC_Cu(II)Pyzha_-5] [29]	Gd(H_2_O)_3_[15- MC_Cu(II)Tyrha_-5] [37]
M-O(oxime)	2.387(7)–2.506(8)	2.412(8)–2.543(8)	2.429(8)–2.451(7)	2.406(5)–2.440(7)
Cu-O(oxime)	1.911(7)–1.935(8)	1.915(8)–1.941(9)	1.912(8)–1.928(7)	1.908(5)–1.944(6)
Cu-O(carbonyl)	1.905(8)–1.961(8)	1.916(8)–1.952(8)	1.935(7)–1.951(7)	1.909(7)–1.959(9)
Cu-N(imine)	1.903(9)–1.914(10)	1.898(10)–1.912(10)	1.906(8)–1.947(10)	1.866(9)–1.904(6)
Cu-N(amine)	1.990(9)–2.021(9)	2.000(9)–2.027(10)	2.006(9)–2.024(10)	1.990(9)–2.037(9)
Cu-O(solv)	2.48(1), 2.57(2)	2.42(1), 2.56(1)	2.16(2)–2.37(1)	2.553(9)
M-O(OH/H_2_O)	2.106(8)	2.047(7)	2.555(10)	2.35(1)–2.54(2)
Cu-O(NO_3_)	2.44(1), 2.88(1)	2.425(8), 2.95(1)	2.32(2)–2.403(8)	2.477(7), 2.95(1)
Cu-O(Tyrha)	2.762(8)	2.655(8)	-	2.825(7)
M...M (across the dimer cavity)	7.1742(6)		-	8.3933(6)
M...M (between dimers)	9.2437(8)		-	8.5158(6)
O(oxime)-Cu-N(imine)	90.8(4)–91.0(4)	90.6(4)–91.5(4)	89.5(3)–90.9(4)	89.3(3)–90.9(4)
O(oxime)-M-O(oxime)	70.6(3)–73.8(3)	71.0(3)–72.9(3)	71.5(3)–72.9(3)	70.7(2)–71.5(2)

**Table 2:** Optimized interatomic distances and topological parameters at the (3,-1) critical points of the covalent bonds in the [Bi(15-MC_Cu(II)Tyrha_-5)]^3+^/[La(15-MC_Cu(II)Tyrha_-5)]^3+^ complexes. The level of DFT calculations is SR-PBE/rL2.

Atoms	Distance, Å	ρ(r_c_), a.u.	∇^2^ρ(r_c_), a.u.	ε
C-C ^1^	1.517–1.523/	0.250–0.252/	–(0.508–0.494)/	0.106–0.107/
	1.518–1.523	0.249–0.252	–0.014	0.104–0.105
C-N_am_^2^	1.494–1.498/	0.244–0.246/	–(0.485–0.475)/	0.016–0.019/
	1.495–1.500	0.244–0.246	–0.011	0.016–0.018
C-N_im_^2^	1.323/	0.358–0.359/	–(1.177–1.172)/	0.277–0.279/
	1.317–1.318	0.362	–0.006	0.287–0.289
C-O	1.283–1.284/	0.361–0.362/	–(0.862–0.850)/	0.104–0.107/
	1.285–1.286	0.36	–0.011	0.099–0.102
N-O	1.381–1.382/	0.323–0.324/	–(0.131–0.128)/	0.069–0.070/
	1.395–1.396	0.311–0.312	–0.003	0.059–0.060
C-C_R_^3^	1.549–1.552/	0.227–0.228/	–(0.380–0.375)/	0.018–0.021/
	1.547–1.550	0.228–0.230	–0.008	0.018–0.021
C-C_ring_^4^	1.508–1.509/	0.248/	–(0.495–0.494)/	0.044–0.049/
	1.508–1.510	0.247–0.248	–0.003	0.043–0.048

^1^ C-C is the bond between the carbon atoms of the hydroximate chelate. ^2^ Herein after indices “am” and “im” correspond to the amine and imine nitrogen atoms respectively. ^3^ C-C_R_ is the bond between the hydroximate chelate and the R = CH_2_(C_6_H_4_)OH substituent. ^4^ C-C_ring_ is the bond between the CH_2_ and (C_6_H_4_)OH fragments.

**Table 3:** Optimized interatomic distances, topological, and energy parameters at the (3,–1) critical points of the coordination bonds in the [Bi(15-MC_Cu(II)Tyrha_-5)]^3+^/[La(15-MC_Cu(II)Tyrha_-5)]^3+^ complexes. Table 2.

Atoms	Distance, Å	ρ(r_c_), a.u.	∇^2^ρ(r_c_), a.u.	*V*(r_c_), a.u.	*E*_int_, kcal/mol	ε
Bi-O_ox_^1^/	2.433–2.442/	0.049–0.050/	0.167–0.170/	–(0.053–0.052)/	16.3–16.8/	0.182–0.184/
La-O_ox_^1^	2.432–2.438	0.056–0.057	0.187–0.189	–0.001	19.8–20.2	0.240–0.241
Cu-O_ox_^1^	1.973–1.984/	0.084–0.086/	0.381–0.396/	–(0.129–0.124)/	38.8–40.4/	0.047–0.048/
	1.992–2.006	0.080–0.082	0.352–0.369	–0.006	35.9–37.7	0.041–0.042
Cu-O_carb_^1^	1.933–1.936/	0.094/	0.444–0.447/	–(0.150–0.148)/	46.5–47.0/	0.014–0.018/
	1.934–1.937	0.094	0.442–0.446	–0.001	46.3–46.9	0.023–0.027
Cu-N_im_	1.890–1.892/	0.114–0.115/	0.474–0.477/	–(0.195–0.194)/	60.9–61.3/	0.071–0.074/
	1.896–1.897	0.113	0.464–0.468	–0.191	59.9–60.1	0.071–0.074
Cu-N_am_	2.034–2.049/	0.083–0.086/	0.281–0.293/	–(0.121–0.115)/	36.0–37.9/	0.020–0.023/
	2.036–2.054	0.083–0.086	0.277–0.292	–0.007	35.4–37.6	0.019–0.021

^1^ Herein after indices “ox” and “carb” correspond to the oxime and carbonyl oxygen atoms, respectively.
